# Exosome‐mediated gut–brain axis signaling in neurodegenerative diseases: Mechanisms, experimental evidence, and therapeutic perspectives—A narrative review

**DOI:** 10.1002/ame2.70226

**Published:** 2026-05-26

**Authors:** Waheeb Sami Aggad, Rakesh Ghosh, Hailah M. Almohaimeed, Zuhair M. Mohammedsaleh, Fayez M. Saleh, Amany I. Almars, S. Renuka Jyothi, Rajashree Panigrahi, Ajoy Kumer, Bikram Dhara

**Affiliations:** ^1^ Division of Anatomy, Department of Basic Medical Sciences, College of Medicine University of Jeddah Jeddah Saudi Arabia; ^2^ Department of Biotechnology & Bioinformatics Northeastern Hill University Shilong India; ^3^ Department of Basic Science, College of Medicine Princess Nourah Bint Abdulrahman University Riyadh Saudi Arabia; ^4^ Department of Medical Laboratory Technology, Faculty of Applied Medical Sciences University of Tabuk Tabuk Saudi Arabia; ^5^ Department of Medical Microbiology, Faculty of Medicine University of Tabuk Tabuk Saudi Arabia; ^6^ Molecular Microbiology and Infectious Diseases Research Unit University of Tabuk Tabuk Saudi Arabia; ^7^ Department of Medical Laboratory Sciences, Faculty of Applied Medical Sciences King Abdulaziz University Jeddah Saudi Arabia; ^8^ Editome: Precision Gene Editing Unit, King Fahad Medical Research Center King Abdulaziz University Jeddah Saudi Arabia; ^9^ Department of Biotechnology and Genetics, School of Sciences JAIN (Deemed to Be University) Bangalore India; ^10^ Department of Microbiology, IMS and SUM Hospital Siksha ‘O’ Anusandhan (Deemed to Be University) Bhubaneswar India; ^11^ Department of Chemistry IUBAT‐International University of Business Agriculture & Technology Dhaka Bangladesh; ^12^ Department of Microbiology, Saveetha Medical College and Hospital Saveetha Institute of Medical and Technical Sciences Chennai India

**Keywords:** Alzheimer's disease, blood–brain barrier, exosomes, extracellular vesicles, gut–brain axis, microRNA, neurodegenerative diseases, neuroinflammation, Parkinson's disease

## Abstract

The stomach and the brain are connected by a sophisticated two‐way communication mechanism called the gut–brain axis. Extracellular vesicles, particularly exosomes, that move bioactive substances between the stomach and the brain, such as proteins, lipids, metabolites, and microRNAs, may improve the gut–brain axis. In the past years, the role of exosome‐mediated communication has been recognized as significant in relation to the etiology, continued progression, and potential treatment of neurodegenerative disorders. The authors of this review article present a summary of the current understanding of the relationship of gut microbiome, exosome biogenesis, and the pathophysiological development of neurodegenerative diseases. Evidence from laboratory studies, animal studies, and newly emerging human studies suggests that microbiome‐based metabolites and inflammatory mediators may modulate how exosomes are produced, what they carry, and how they interact with the blood–brain barrier. These exosomal signals may impact neuroinflammation, neuronal signaling, and the spread of pathological proteins of neurodegenerative diseases, such as Alzheimer's disease, Parkinson's disease, amyotrophic lateral sclerosis, and Huntington's disease. In addition, they examine some possible ways to target the gut–brain axis from a therapeutic perspective, including manipulating the gut microbiome, providing probiotics and/or prebiotics, performing fecal microbiota transplantation, and/or using engineered extracellular vesicles as vehicles for drug delivery. The authors also outline some of the methodological differences that make it difficult to assess the effects of exosomes.

## Graphical abstract

The gut–brain axis is a bidirectional communication network between the intestines and brain, mediated by gut microbiota and exosomes, that regulates neuroinflammation, protein aggregation, and neuronal health processes central to neurodegenerative diseases. Therapeutic interventions, including dietary modification, probiotics, prebiotics, and fecal transplants, show promise, though significant research gaps remain regarding microbiota–exosome interactions and the development of safe, effective treatments before clinical translation can be achieved.

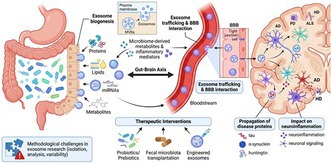

## 1 INTRODUCTION

**FIGURE 1:**
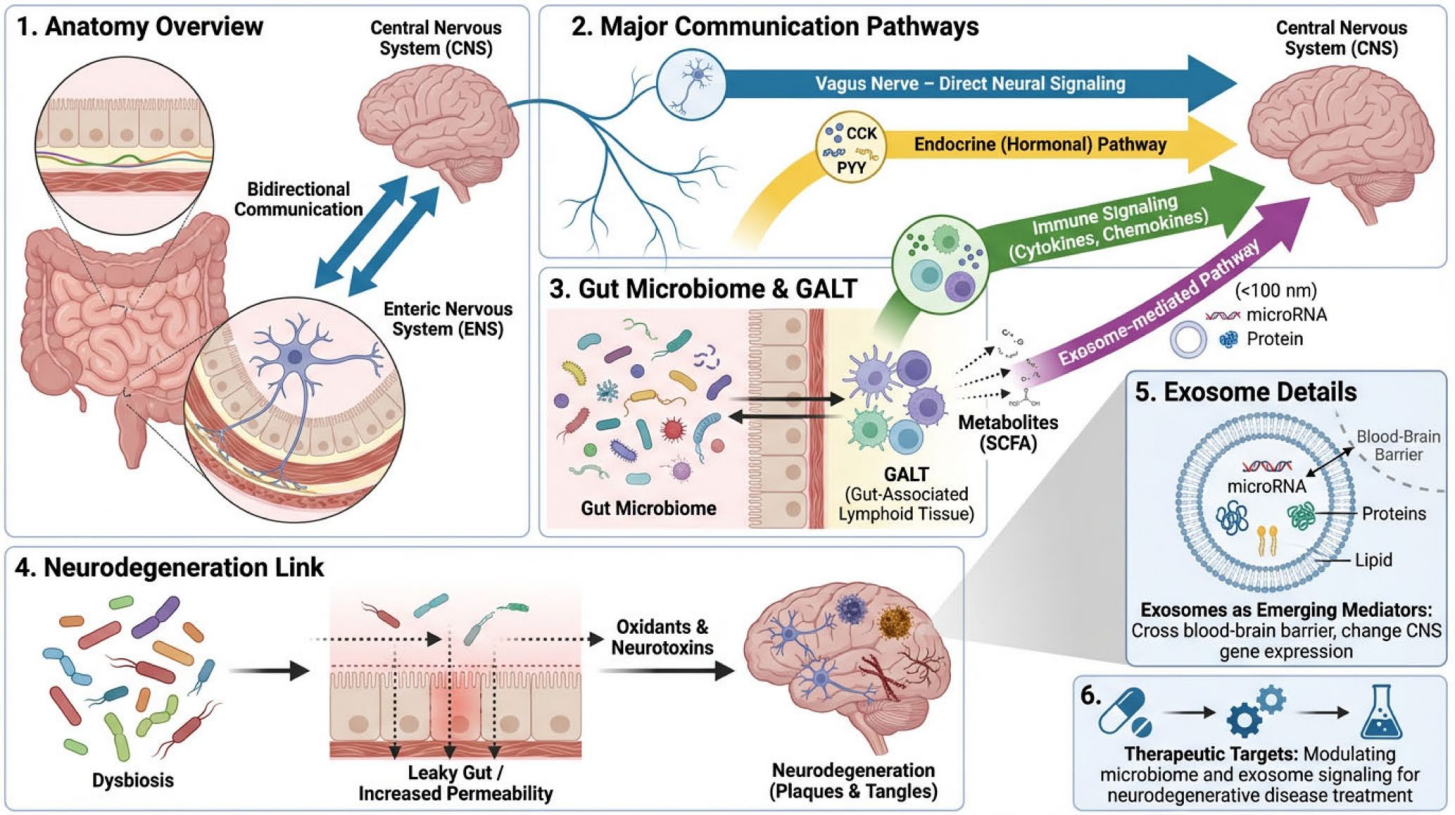
Intestinal immune system and mucosal homeostasis of gut microbiome (created with BioRender.com).

The gut–brain axis is a bidirectional communication and integration framework that unites the central nervous system (CNS) with the enteric nervous system (ENS), a group of neurons within the lining of the gastrointestinal tract.^1^ The gut–brain axis consists of a unique wiring system that enables ongoing communication between the gut and brain, modulating multiple physiological and behavioral processes through numerous pathways that may be overlapping or interacting. One of the important pathways for communication^2^ is the vagus nerve, because it provides the quickest connection for signaling to the brain with a direct link. Another pathway is through endocrine signaling using hormones or neurotransmitters that are produced in either the gut or brain. Gut hormones like cholecystokinin (CCK) and peptide YY (PYY) that are released into the brain can provide signaling.^3^ Immune pathways are incredibly relevant when considering how the cytokines and chemokines in the gut might affect the neuroimmune function along the gut–brain axis. Recent studies have demonstrated that gut‐associated lymphoid tissue (GALT), a prime constituent of the immune system (see Figure 1), can influence immune signaling in the gut, which may influence signaling along the gut–brain axis (GBA).

The GALT contains a large concentration of immune cells, which constantly interact with gut microbes and facilitate informed immune responses, both locally and systemically, to influence brain function.^4^ The gut microbiome is an enormous network of living microbes, which coexist within the gastrointestinal tract. This collective microbial community makes up an integral piece of the GBA. The constituents and variety of these gut microbes systematically shape how healthy and functioning a person's brain will be.^5^ Eliminating these gut microbes or causing imbalances in gut microbe‐community structures may lead to the development of many different types of neurodegenerative diseases. Mechanisms linking gut dysbiosis to neurodegeneration have been proposed in the literature and include increased permeability of intestines due to chronic inflammatory processes, production of oxidants, and production of neurotoxins.^6^ Certain gut microbial taxa have been shown to be significant in the development of neurodegenerative diseases; for instance, individuals with Alzheimer's disease exhibit higher levels of Bacteroidetes than do health controls.^7^ Because gut microbes produce a wide range of metabolites, such as short‐chain fatty acids (SCFAs), their products may also play a role in brain involvement.^8^ Recent publications have highlighted that gut–brain communication is predominantly through exosomes, which are vesicles that originate from cells and are < 300 nm.^9^ Extracellular vesicles (EVs) are made of proteins, lipids, and nucleic acids (i.e., microRNAs), and represent an efficient evolutionary strategy for cells to communicate with one another and engender change in the recipient's phenotype (by passing their protein content). Exosomes are able to relay information from gut signaling molecules to the brain because they typically have diameters of less than 100 nm and can therefore more easily cross the blood–brain barrier (BBB).^10^ Emerging data suggest that exosomal microRNAs are key mediators of cell‐to‐cell signaling from the gut to the CNS, as they can change gene expression and modify several other cellular events within the brain. In addition, the gut microbiome will induce alterations in the production of exosomes and different cargo contained within the exosomes, further entrenching gut–brain communication. Notably, the GBA is a complex communication network that encompasses neural, endocrine, immune, and microbial components.^1^ The gut microbiome will have radically different effects on brain health/function via a range of possible signaling processes. Importantly, exosomes (with microRNAs being one of the many particles contained within) have recently been identified as a new signaling player in gut–brain communication from the gut to the brain.^9^ Understanding the intricate interplay between the gut microbiome, exosomes, and the brain is crucial for developing novel therapeutic strategies for neurodegenerative diseases. There has been a growing interest in the communication between the gut and the brain over the past several years. However, how exosome‐mediated signaling participates in the progression of neurodegeneration has not yet been fully elucidated. Past studies have focused primarily on the classical forms of communicating, such as the nervous system, the immune system, and the endocrine system, whereas the role of EVs in long‐range molecular transport has only recently begun to be studied. The literature in this area is full of studies of microbiomes, of the biology of EVs, and the models of neurodegenerative diseases that do not integrate these disparate areas into a holistic understanding. Therefore, this narrative review seeks to review the current literature on the role of gut microbiota in regulating the production and cargo composition of exosomes, the involvement of exosomes in gut–brain signaling, and how these processes may contribute to the pathogenesis and treatment of neurodegenerative diseases.

## 2 MATERIALS AND METHODS

This article was prepared based on the comprehensive analysis of scientific literature related to GBA, metabolites produced by bacteria, and cellular messenger roles of exosomes in the pathogenesis of neurodegenerative disorder syndromes. Studies identified through electronic databases (PubMed, Scopus, Web of Science, and Google Scholar) were evaluated until December 2024 that focused on the role of exosome‐mediated signaling between intestinal microbiota and the CNS as mediated by specific pathways, including “GBA,” “exosomes,” “microbiota,” “metabolite from gut bacteria,” neurodegenerative disorders (such as Alzheimer's disease and Parkinson's disease), and intercellular signal communication via exosomal transport. By combining keywords in various combinations (AND/OR) using Boolean logic and syntax, studies most pertinent to exosome‐based signaling were identified.^11^ Peer‐reviewed journal articles, review articles, and experimental studies discussing biochemical pathways linking intestinal microbiota, formation of exosomes, and progression of neurodegeneration were included; non‐peer‐reviewed articles, abstract‐only reports (no full‐text available), studies without a clear relation to GBA/exosome physiology were excluded. The data presented in the literature reviewed were analyzed based on the current understanding of exosomal biochemical pathways involving exogenous (externally derived/external source) biochemical substances, with peer‐reviewed literature used to support the relevance of the proposed therapeutic agents or methods used to diminish/stop the process of neurodegeneration.

## 3 IMPACT OF GUT MICROBIOTA ON EXOSOME PRODUCTION AND FUNCTION

### 3.1 Microbial metabolites and exosome biogenesis

**FIGURE 2:**
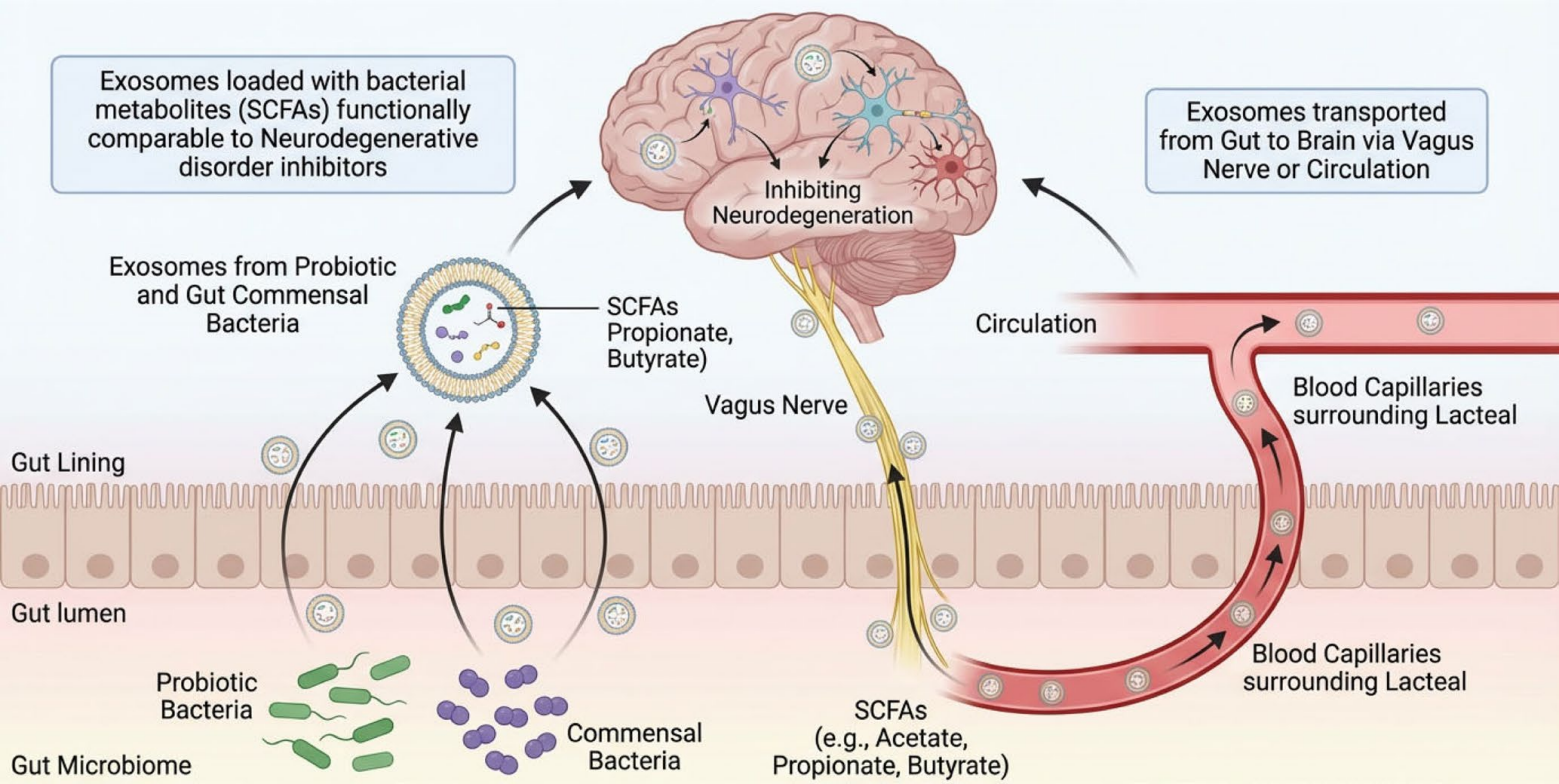
Exosomes produced by gut‐inhabiting probiotic and commensal bacteria contain short‐chain fatty acids (SCFAs), which may inhibit neurodegeneration (created with BioRender.com).

The gut microbiota plays a major role in regulating exosome production and function with respect to their biogenesis, cargo, and, ultimately, their function in intercellular communication. The mechanism underlying this role is complex, with the gut microbiota influencing exosome production through the production of metabolites, as well as through effects on host cell processes and direct interactions with the host cells. Probably the greatest impact of the gut microbiota on exosome production is mediated through metabolites produced by gut bacteria, such as SCFAs like butyrate, propionate, and acetate, which are produced from the fermentation of dietary fibers.^12^ These SCFAs can either influence the production and biogenesis of exosomes and release process or can be present in bacterial surface‐derived exosome vesicles (Figure 2). Butyrate appears to enhance the release of exosomes from intestinal epithelial cells and may do so through the modulation of intracellular signaling pathways or change in the appearance of genes involved in the process of exosome biogenesis.^13^ Other microbial metabolites can also affect exosome production. Bile acids altered by gut microbiota can be associated with exosomal release from hepatic cells, whereas microbial metabolites of tryptophan (e.g., indole) can modulate exosome production from all cell types.^14^

Recent research has suggested that lipopolysaccharides (LPS) from gut microbes play a role in exosome biogenesis.^15^ LPS is a pattern recognition molecule found on the outer membrane of Gram‐negative bacteria, which can induce an inflammatory reaction and promote exosomal release from immune (and possibly all) cell types.^16^ Perhaps this also explains the higher levels of exosome release in diseases of gut dysbiosis and inflammation. The gut microbiota not only produces exosomes, directly or indirectly, but can also modify the cargo type encapsulated in exosomes. Gut microbes can also alter the proteins encapsulated in exosomes, thus changing the signaling ability of exosomes. Some bacteria can promote the addition of specific proteins to exosomes, which are then taken up by recipient cells and thus change the function of the recipient cell.^17^ The gut microbiota also broadly changes the miRNA content of exosomes.^18^ miRNAs are noncoding RNA molecules that regulate gene expression, and their presence within exosomes impacts distant cells.^19^ Gut microbes can also influence the host cell miRNA expression, which can further alter the miRNA content of exosomes. These exosomal miRNAs, once released, can also be transferred to other tissues, including the brain, presumably altering gene expression and cellular processes. Recent work has identified that gut microbiota elicits changes in the lipid composition of exosomes. These changes in lipid composition can alter exosome stability and impact uptake by recipient cells, which could directly influence the functional nature of exosomes.^20^

### 3.2 Microbial regulation of exosome cargo

Gut microbiota is an important modulator of exosomal biogenesis and function. The gut microbiota modulates exosome production through changes to host metabolic output and cellular processes, and can also directly interact with host cells to alter exosomal biogenesis, cargo, and even influence intercellular signaling. The relationship between gut microbiota and exosomes has significant implications for numerous physiological processes, including those at the GBA and the orchestrating pathophysiology of neurodegenerative diseases.^21^ It is critical to recognize the complex interplay between gut microbes and exosomes when developing new therapeutic approaches targeting the GBA to promote brain health or treat neurodegenerative diseases.

### 3.3 Exosomes and the transfer of signals from the gut to the brain

Exosomes play a key role in sending signals regarding gut function to the brain, as they contain bioactive molecules that can regulate neuronal activity and influence biological processes. These vesicles are produced by the lining of the intestine as well as by probiotics and gut bacteria. The exosomes that are produced by the intestines enter the bloodstream and can pass through the BBB, thereby transferring their contents to cells in the brain.^21^ This means that communication between the gut and the brain, through exosomes, is one of the pathways by which gut microbes influence brain function, which can ultimately contribute to health and disease. Exosomes transmit information about microbial metabolites, as well as their own metabolic signals, from the gastrointestinal tract back to the brain: for example, SCFAs generated from the fermentation of dietary fiber by microorganisms.^12^ The three primary SCFAs produced by gut microbiota include butyric acid, propionic acid, and acetic acid, all of which have been shown to affect brain function through neuroinflammation, oxidative stress, and neurotransmitter production.^8^

**FIGURE 3:**
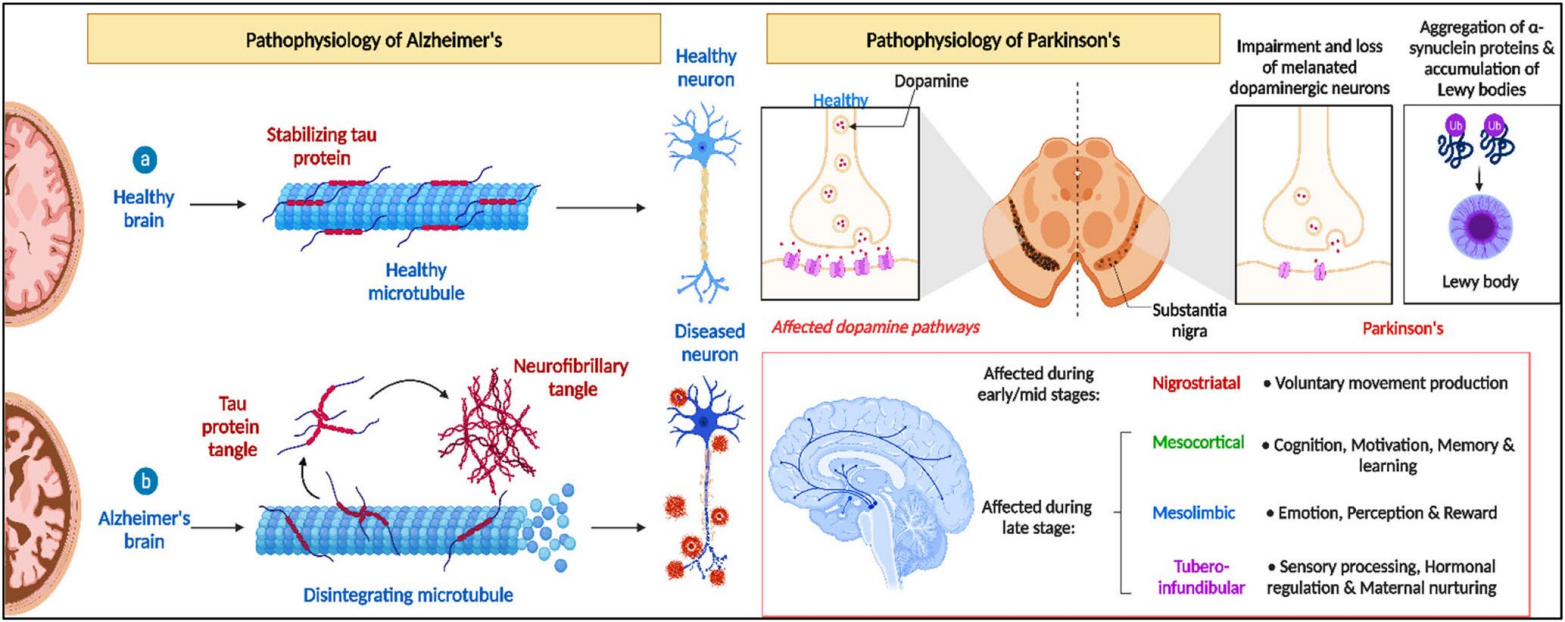
Pathophysiology of Alzheimer's disease and Parkinson's disease (created with BioRender.com).

Exosomes may also mediate the transfer of additional microbial metabolites such as kynurenine and indole, both of which are produced from tryptophan metabolism that has been assessed in regard to mood, mood modulation, and neuroinflammation.^22^ Furthermore, several studies have recently provided preliminary support for exosomal transfer of gut‐derived neurotoxins such as amyloid‐β, a protein believed to play an important role in the pathophysiology of Alzheimer's disease^23^ (see Figure 3), which may be involved in the development of neurodegenerative diseases and very likely facilitates the dissemination of neurodegenerative diseases. Exosomes may also mediate the communication of immune signals from the gut to the brain, as they contain gut‐derived cytokines and chemokines. Cytokines and chemokines are crucial mediators of inflammation and the immune response; thus the exosomal transfer of gut‐derived immune signals is likely to alter pathways of neuroinflammation. Given that gut‐derived exosomes contain pro‐inflammatory cytokines such as interleukin 1β (IL‐1β) and tumor necrosis factor α (TNF‐α), they could also alter neuroinflammation,^24^ and neuroinflammation represents a common aspect of one or more pathways of neurodegenerative diseases. Exosomes also possess a potential for mediating the transfer of pathogen‐associated molecular patterns (PAMPs), which are molecules created by microbes that can activate immune cells.^25^ The exosome transfer of PAMPs is likely to activate neuroimmune processes and exacerbate neuroinflammation.

In addition, exosomes permit the transfer of neurotransmitters and other neuroactive molecules that may activate neuronal activity and neuronal communication. It has been speculated that exosomes can transfer neurotransmitters associated with mood and cognition; specifically, exosomes can transfer GABA and serotonin to the CNS.^26^ Exosomes are also speculated to mediate the transfer of neurotrophic factors, particularly brain‐derived neurotrophic factor (BDNF), to promote neuronal growth, survival, or differentiation.^27^ Trafficking of neuroactive molecules, such as neurotransmitters and neurotrophic factors, via exosomes has potential to alter brain function and help maintain neuronal health. Furthermore, more recently, the role of exosomes in the gut–brain serotonin axis has been discussed, and gut‐derived exosomes can influence serotonin signaling in the brain. Therefore, exosomes are an important mediator of gut‐to‐brain signaling and signal diverse molecules to impact brain function. Exosomes facilitate communication by delivering microbial metabolites, immune signals, and neuroactive molecules. Therefore, exosomes represent a way by which the gut microbiota communicates with the brain (the GBA) and physiologically influences a number of different biological processes through exosome‐mediated communication. This pathway of exosome communication is highly relevant to referencing a part of the gut–brain pathway's role in health and disease, specifically neurodegenerative disorders. Future studies are imperative to better expose the mechanisms by which exosomes communicate and their potential metabolic, functional, and neuromodulatory effects on the brain, to aid in the development of future therapeutic strategies that may promote brain health and/or ameliorate the consequences associated with neurodegeneration.

### 3.4 Exosomes and neurodegenerative disease pathogenesis

Exosomes are increasingly recognized as important mediators of neurodegenerative disease pathogenesis, contributing to their pathogenesis and progression through multiple biological mechanisms. These nano‐sized vesicles are released from cells in the brain as well as other tissues, including the gut that can carry various cargo, including proteins, lipids, and nucleic acids, all of which have the potential to affect neuronal function and contribute to neurodegeneration. One of the critically important mechanisms exosomes contribute to neurodegenerative disease pathogenesis is through the induction of neuroinflammation.^28^ Gut dysbiosis results in an imbalance in the gut microbial community that can cause increased gut permeability, leading to the systemic release of pro‐inflammatory molecules, including LPS.^29^ The LPS and gut microbiota may induce gut‐level immune responses and release exosomes, carrying inflammatory signals from the gut and immune cells, to other organ tissues, including the brain. These exosomes could traverse the BBB, carrying the inflammatory signals from the gut and immune cells and contributing to neuroinflammation.^28^

Exosomes may also contribute to the activation of microglia, the resident immune cells in the CNS.^30^ When activated, microglia can secrete large amounts of pro‐inflammatory cytokines and chemokines but can also trigger neuroinflammation that can ultimately contribute to neuronal injury. Exosomes and resulting neuroinflammation have been documented in other neurodegenerative diseases, including Alzheimer's disease and Parkinson's disease (Figure 2). Besides initiating neuroinflammation, exosomes can contribute to neurodegeneration, because they assist in the transmission of misfolded proteins, which are the primary pathological characteristics that underlie neurodegenerative diseases. For example, in Alzheimer's disease, exosomes may contain amyloid‐β, the protein responsible for both aggregation and plaque formation in the brain, thereby contributing to a transactive cycle that results in neuronal death.^23^ Exosomes can move amyloid‐β, for example, in the space between concurrent neurons or glial cells in the brain. This process can contribute to the spread of Alzheimer's disease. In Parkinson's disease, exosomes may also facilitate the movement of α‐synuclein, another protein associated with aggregation that conspires to increase neuronal death.^31^ Exosomes can mediate the movement of α‐synuclein to/from neurons, facilitating the propagation of para‐α‐synuclein pathology associated with α‐synuclein aggregation, phosphorylation, and aggregation.

**FIGURE 4:**
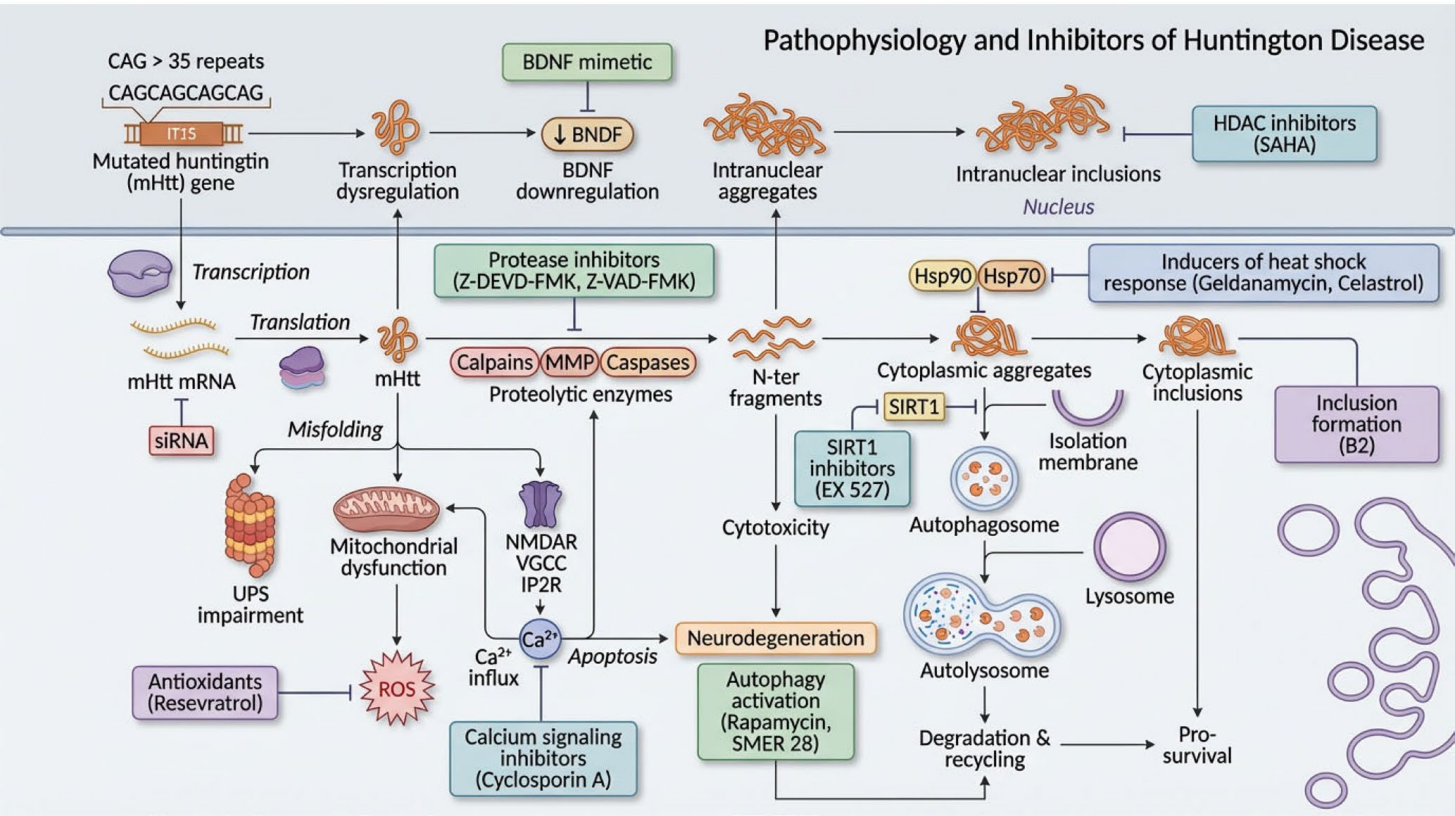
Pathophysiology and pathway inhibitors of Huntington's disease (created with BioRender.com).

Recently, researchers have also documented a role of exosomes in the pathophysiology of other degenerative diseases, including Huntington's disease (HD) and amyotrophic lateral sclerosis (ALS). For example, exosomes are able to transfer the mutant huntingtin in HD. The aggregates formed by mutant huntingtin may cause neuronal dysfunction and incapacitation, although^32^ (Figure 4). Exosomes may provide a novel avenue for the transfer of mutant huntingtin within and among cells, potentially promoting the propagation of disease.^32^ In ALS, exosomes can transfer TDP‐43, the protein that has aggregates in motor neuron cells, and the aggregation ultimately causes degeneration to the parent location.^33^ Thus, exosomes play a significant role in the pathogenesis of neurodegenerative diseases by promoting neuroinflammation, the spread of misfolded proteins, and neuronal dysfunction.

### 3.5 Therapeutic implications of the gut–brain axis

**FIGURE 5:**
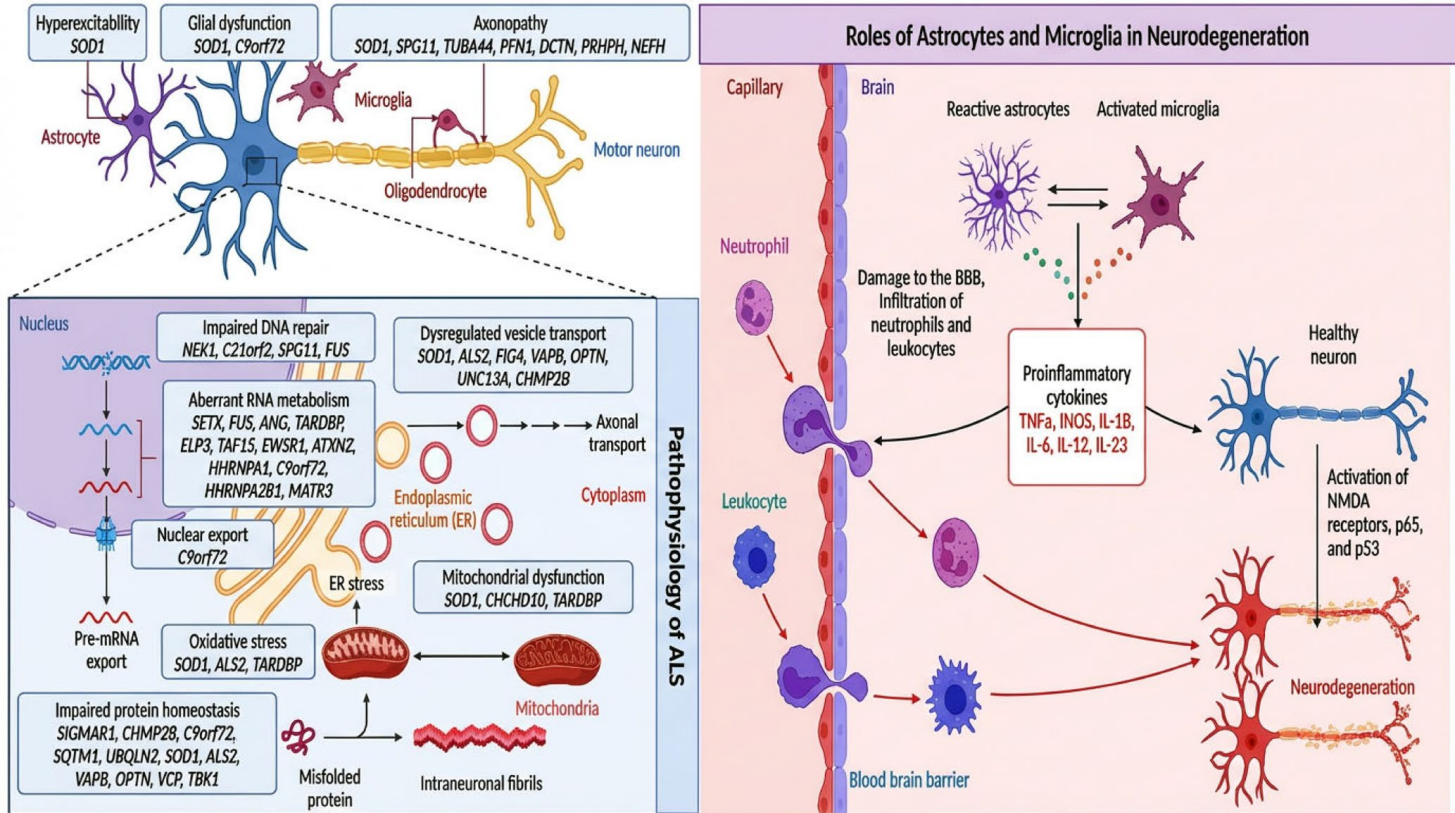
Pathophysiology of amyotrophic lateral sclerosis (ALS) and roles of astrocytes and microglia in neurodegeneration (created with BioRender.com).

**FIGURE 6:**
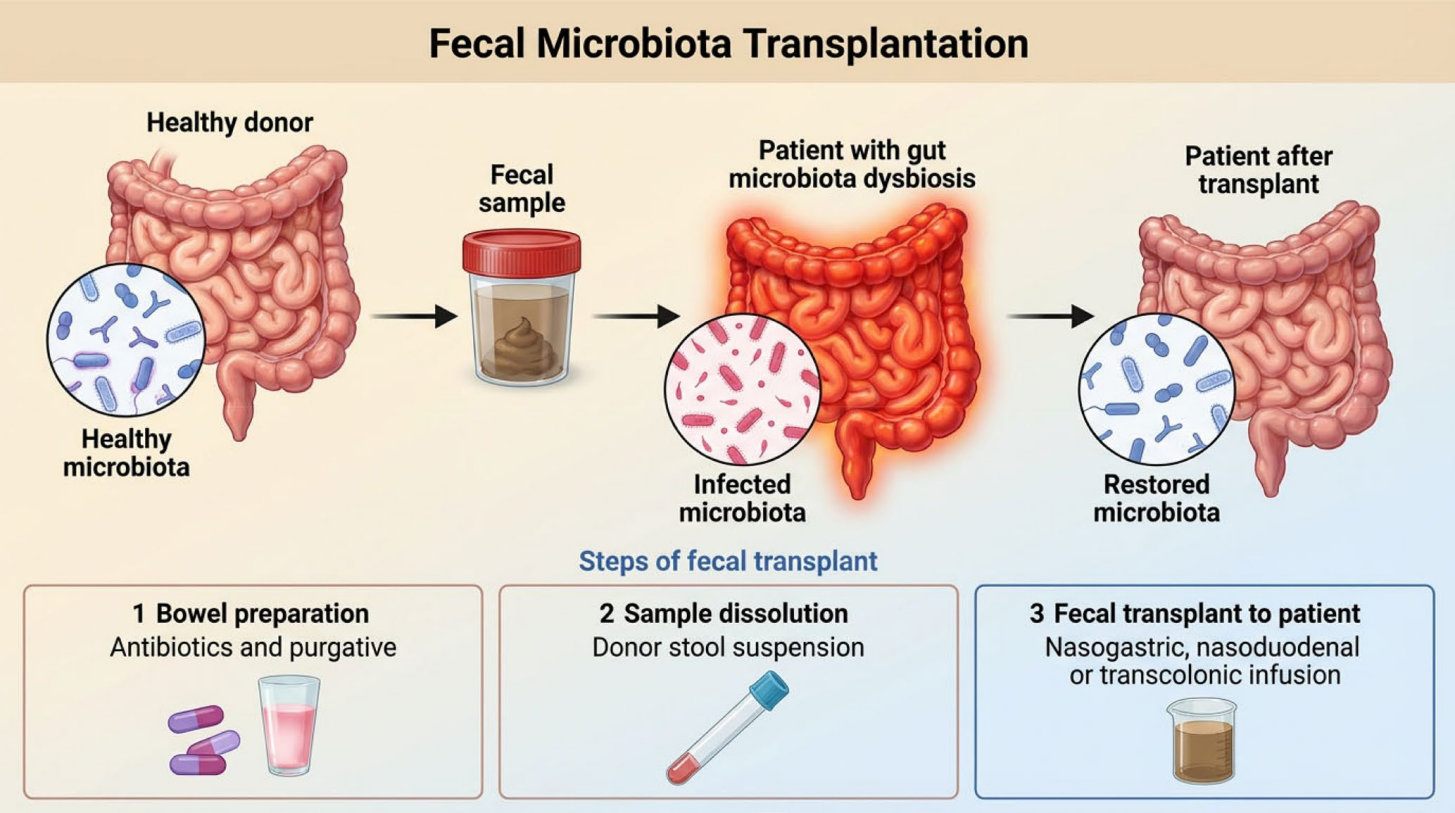
Fecal microbiota transplantation (created with BioRender.com).

The GBA offers interesting new therapeutic possibilities for the treatment of neurodegenerative illnesses because it is a complex communication network with a wide range of possible beneficial effects on brain health and functional outcomes. Exosomes have an intriguing role in the pathophysiology of neurodegenerative illnesses; for example, they may facilitate the progression of TDP‐43 pathology in ALS (Figure 5). By focusing on the gut microbiota and how it interacts with the brain, there are special opportunities to affect the course of disease in ways that may enhance patient outcomes. Modulating the gut microbiota and exosome‐mediated signaling is one broad therapeutic route for putting these tactics into practice. Dietary therapies (e.g., prebiotics, probiotics, and synbiotics) use the gut microbiota to generate the proper dietary components realized as behaviors. Probiotics are live microorganisms that offer health benefits to the host when consumed in appropriate doses; prebiotics are indigestible food ingredients that support the growth of beneficial gut bacteria; and synbiotics combine prebiotics and probiotics for a synergistic effect that enhances the health benefits of both groups.^34^ To change the generation of exosomes and exosome cargo that can send positive signals along the GBA, dietary components can modify the makeup and function of the gut microbiota. Another innovative therapy approach is fecal microbiota transplantation (FMT), which involves transferring fecal material from a healthy donor to a patient with gut dysbiosis^35^ (Figure 6). Reestablishing a healthy gut microbial community is the aim of FMT, which effectively treats a number of illnesses, including *Clostridium difficile* infection.^36^ According to recent research, FMT may be used to treat neurodegenerative diseases. It may do this by changing the gut microbiota and the number of known pathways that connect the gut to the brain.^37^ To further understand the processes and improve the therapeutic benefits of FMT in the treatment of neurodegenerative diseases, more mechanistic research studies utilizing FMT are required. Additionally, personalized microbiome regulation has emerged with the aim of incorporating and accounting for the individual differences in gut microbiota composition and function. To improve unique and targeted treatment responses, it has been difficult to customize the regulation of therapies based on each patient's gut microbiota composition. New developments in precision medicine and microbiome assessments will support the ongoing creation of tailored approaches. Lastly, there is still a push to develop probiotic and prebiotic treatments to control exosome‐mediated interactions that can bidirectionally modify communication along the GBA in addition to modifying gut microbiota.^38^

This includes identifying probiotic strains with exosome‐producing advantages or that trigger host cells to produce beneficial exosomes. Prebiotic approaches include creating beneficial microbial metabolites, such as SCFAs, that may affect exosome production and cargo composition.^39^ In addition, researchers are also investigating the development of probiotics engineered for delivering therapeutics through exosomes as a means of targeted therapy for neurodegenerative diseases.

Another therapeutic approach may directly target the exosome‐mediated communication along the GBA through inhibition of exosome biogenesis or release, halting the production of exosomes with negative signals.^40^ An equally driven line of inquiry might be focused on ways to inhibit exosome uptake at the level of the brain, such that exosomes with harmful cargo are undelivered to the brain cells. Additionally, there has been an interest in engineering exosomes for drug delivery to the brain, using their inherent property of altering the BBB and targeting particular regions of the brain with therapeutics.^41^

The GBA provides exciting opportunities for innovative therapeutic approaches to neurodegenerative diseases. By modifying the gut microbiota, developing targeted probiotic and prebiotic therapies, and targeting exosome‐mediated mechanisms, researchers are developing ways to modify the disease course and improve patient outcomes. Although we may not know everything yet about the complexity of the interactions with the GBA and novel therapeutic strategies, research remains promising for effective treatments for neurodegenerative diseases (Table 1).

**TABLE 1:** Microbiota–exosome‐targeted therapeutic strategies for neurodegenerative diseases.

Strategy	Evidence level	Disease context	Mechanism of action	Key molecular/Cellular targets	Reported or proposed therapeutic outcomes
Probiotics	Preclinical studies and early clinical trials	Alzheimer's disease (AD), Parkinson's disease (PD)	Restoration of gut microbial balance and modulation of microbial metabolite production influencing exosome signaling	SCFAs, inflammatory cytokines, exosomal miRNAs	Reduction of neuroinflammation, improved gut barrier integrity, potential cognitive and motor function improvement
Prebiotics	Mainly preclinical studies	Neuroinflammation, early neurodegeneration	Stimulation of beneficial microbial populations leading to altered metabolite‐mediated exosomal communication	Butyrate, acetate, microbial‐derived metabolites	Modulation of immune responses, decreased neuroinflammatory signaling
Fecal microbiota transplantation (FMT)	Experimental and early‐stage clinical studies	AD, PD	Transfer of healthy microbial communities that may influence host exosome production and immune signaling	Microbial diversity, gut barrier regulators, inflammatory mediators	Restoration of gut microbiota composition, improvement in neurological symptoms in preliminary studies
Engineered exosomes	Preclinical and experimental therapeutic studies	Central nervous system (CNS) disorders, including AD and PD	Use of modified exosomes as delivery vehicles for therapeutic molecules capable of crossing the blood–brain barrier	Therapeutic miRNAs, siRNAs, proteins, small‐molecule drugs	Targeted drug delivery to neural tissues and modulation of pathological signaling pathways
Exosome inhibition	Experimental mechanistic studies	Neuroinflammation and neurodegeneration	Suppression of pathogenic exosome release or uptake to prevent propagation of inflammatory or neurotoxic signals	Exosome biogenesis pathways (e.g., ESCRT components), inflammatory mediators	Potential reduction of inflammatory signaling and slowing of neurodegenerative progression

Abbreviations: miRNA, microRNA; SCFA, short‐chain fatty acids; siRNA, small interfering RNA.

### 3.6 Challenges and future directions

Despite the promising therapeutic applications of the GBA, several difficulties present themselves in the development of clinical applications. One of the most important hurdles is effectively elucidating the complexities of microbiome–exosome interactions. It is understood that gut microbes can impact exosome production and cargo, but what specific microbial factors and pathways that mediate this interaction are not yet fully understood. Effective identification of gut microbes and understanding of how they modulate exosome biogenesis and cargo sorting is necessary before they equip us with target interventions. Moreover, it is necessary to clarify mechanisms that allow exosome uptake into cell types in the brain, and how this influences the delivery of cargo. Addressing these areas is critical for optimizing exosome‐based therapies and their proper use. Another considerable barrier is the design of novel therapeutic interventions that target the GBA. Although there is a lot of exciting results from microbiome modulation via dietary strategies and FMT, further optimizing these strategies into therapeutic benefit is needed (Figure 6). The identification of specific microbes and sites to mitigate or promote brain health, as well as personalized recommendations based on individual microbiome profiles, remains essential.

Moreover, there are many hurdles to developing specific exosome‐based therapies for neurodegeneration disease, such as designing exosomes to achieve drug delivery to the brain, crossing the BBB, and properly targeting areas of concern in the brain. Additionally, where research related to the GBA is transferred to the clinic, ethical and safety considerations are paramount. Here it is required to ensure the safety and efficacy of microbiome‐based treatments, especially in situations involving FMT, with considerations from donor selection to protocol standardization and long‐term monitoring for possible adverse effects; in addition, ethical handling of genetic engineering of microbes and exosomes will require careful consideration. Clear regulations regarding development and application of exosome‐based therapies are an important first step for responsible applications.

Future research directions should include enhanced levels of both in vitro and in vivo models to determine microbiome–exosome interactions, which will allow researchers to interrogate the interplay between gut microbes, exosomes, and the brain, to identify therapeutic targets and generate interventions.

Microbiome modulation with exosome‐based treatments is one such promising way to enhance therapeutic outcomes using both gut and exosome‐mediated communication routes. In addition to modulation of the microbiome, exosomes may be a promising new avenue to investigate as biomarkers for neurodegenerative diseases. Exosomes derived from biofluids (such as blood and cerebrospinal fluid) may contain disease‐specific molecules to enable early diagnosis, tracking disease progression, and monitoring treatment response. Ultimately, there are ongoing challenges in translating GBA research into clinical outcomes; however, the area of GBA research holds great promise in the future development of novel therapeutic approaches to neurodegenerative diseases. Admittedly, there is significant work to be done to address these challenges, and the future approaches listed above aimed at harnessing the complete therapeutic potential of the GBA need to be followed up. In conclusion, in the future, it would be expected for researchers to maximize the therapeutic opportunities available to individuals living with neurodegenerative diseases.

Future studies should prioritize (i) standardization of EV isolation and characterization protocols, (ii) improved in vivo tracking technologies for exosome trafficking, (iii) differentiation between host‐derived and microbiota‐derived vesicles, and (iv) longitudinal clinical studies investigating exosomal biomarkers in neurodegenerative disease progression.

## 4 CONCLUSION

The GBA is a rapidly advancing area of research in the field of neurodegenerative disease. The GBA integrates bidirectional communication among neural, endocrine, immune, and microbial pathways to impinge on both the health and functions of the brain. The gut microbiome (the community of microorganisms that resides in the gastrointestinal tract) is a major participant in the GBA and, importantly, modulates brain function by producing metabolites and other substances, as well as by regulating immune signaling and interactions with the enteric nervous system. Recently, exosomes have been identified as critical mediators of gut–brain communication. Exosomes are nano‐sized vesicles released by all cell types, including bacteria, and they carry proteins, lipids, and nucleic acids, including microRNAs. Exosomes can transfer their cargo to responding cells to alter their function. Exosomes have been reported to interact with the BBB through several mechanisms, including receptor‐mediated uptake, transcytosis, and interactions with endothelial cells. However, their ability to cross the BBB depends on multiple factors such as vesicle origin, surface proteins, inflammatory state of the barrier, and physiological conditions.

The emerging research path is to study the interrelationships between the gut microbiome and exosomes. Gut microbes affect exosome production and their cargo delivery, including their signaling capabilities and potential neurological effects. This signifies an important connection in the field of the GBA, potential role in health‐disease connections, and obviously neurodegenerative studies. Gut dysbiosis has been associated with the initiation and progression of many neurodegenerative diseases; exosomes might be the vehicle for the harm or consequences of gut dysbiosis. Interestingly, the GBA represents a tremendous opportunity to intervene in neurodegenerative diseases via therapeutic application. Through diet, probiotics, prebiotics, and fecal microbiota transplantation, there is a concerted effort to change the gut microbiome back to a healthy microbial community and to initiate beneficial signaling along the GBA. Additionally, targeting exosome‐mediated interactions via inhibition of biogenesis, blocking uptake, and engineering exosomes for drug delivery represents a new therapeutic opportunity. There is still a substantial gap in understanding the GBA to better translate it into clinical practice and fulfill its clinical promise, including understanding the GBA and exosome attributes. Additional research is necessary to further comprehend the specific mechanisms controlling the relationship between microbiome and exosomes, to determine the treatment options involved, and to address ethical and safety considerations.

**FIGURE 7:**
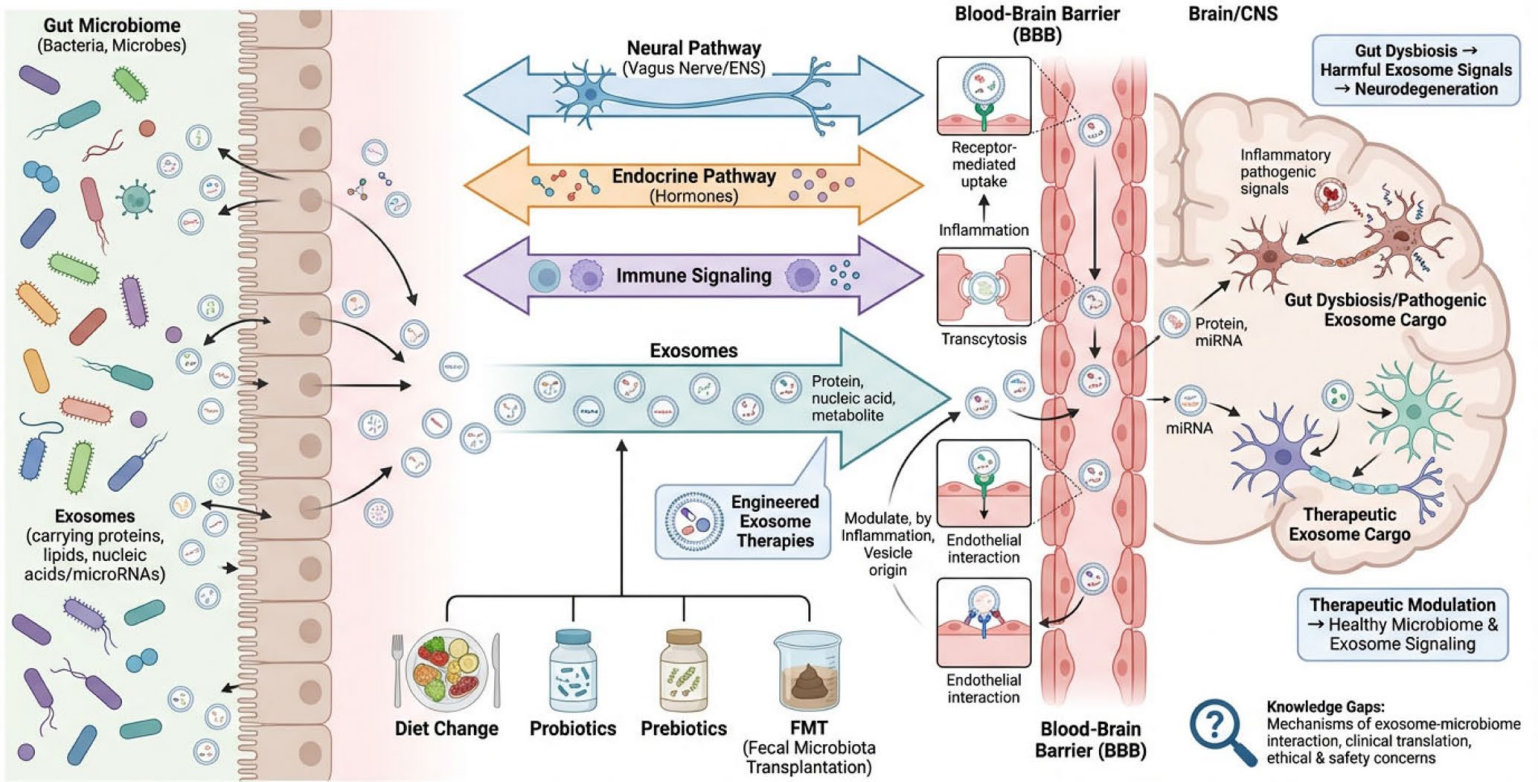
Mechanistic overview of the gut–brain axis in neurodegenerative disease pathogenesis.

The GBA and the relationships between the gut microbiome, exosomes, and the brain constitute a crucial area of investigation for neurodegenerative disease research (Figure 7). Continued exploration of the GBA and microbiome–exosome interactions will enable researchers to understand the therapeutic aspects and begin a pathway toward treatments that positively impact the lives of patients and manage the changes seen in neurodegenerative disease. To achieve this, it will be critical for researchers to seek collaboration across diverse areas of expertise, include or develop robust experimental models, and acknowledge the ongoing ethical and safety issues related to modulating GAB activity. Future directions in neurodegenerative disease research lie in leveraging the underlying biology of the GBA to develop treatments that target the causes of deficits experienced by patients with neurodegenerative disorders. A large body of evidence is now supporting the notion that exosome mediation is an important part of the ongoing exploration of the communication that takes place between the gut and the brain (the GBA). Although a growing number of experimental studies support the role of EVs in exchanging inflammatory signals, metabolites, and pathogenic proteins between the gut and the brain, there are still many missing pieces regarding the precise mechanisms of EVs, and how this knowledge can be translated into new clinical therapies. To make progress in using this mechanistic knowledge to develop new clinical therapies, it will be necessary to conduct interdisciplinary studies involving microbiome science, EV biology, and neurodegenerative disease models.

## AUTHOR CONTRIBUTIONS

**Waheeb Sami Aggad:** Conceptualization; data curation; formal analysis; funding acquisition; investigation; methodology; project administration; resources; software; supervision; validation; visualization; writing – original draft; writing – review and editing. **Rakesh Ghosh:** Conceptualization; data curation; formal analysis; investigation; methodology; software; validation; writing – original draft; writing – review and editing. **Hailah M. Almohaimeed:** Conceptualization; data curation; formal analysis; funding acquisition; methodology; resources; validation; writing – original draft; writing – review and editing. **Zuhair M. Mohammedsaleh:** Conceptualization; data curation; formal analysis; funding acquisition; investigation; methodology; resources; validation; visualization; writing – original draft; writing – review and editing. **Fayez M. Saleh:** Data curation; formal analysis; funding acquisition; investigation; methodology; resources; software; writing – original draft; writing – review and editing. **Amany I. Almars:** Data curation; formal analysis; funding acquisition; investigation; methodology; resources; software; validation; writing – original draft; writing – review and editing. **S. Renuka Jyothi:** Conceptualization; data curation; formal analysis; investigation; methodology; software; writing – original draft; writing – review and editing. **Rajashree Panigrahi:** Conceptualization; formal analysis; investigation; methodology; resources; software; writing – original draft; writing – review and editing. **Ajoy Kumer:** Conceptualization; data curation; funding acquisition; investigation; methodology; project administration; software; supervision; validation; writing – original draft; writing – review and editing. **Bikram Dhara:** Conceptualization; data curation; formal analysis; investigation; project administration; resources; software; supervision; validation; visualization; writing – original draft; writing – review and editing.

## FUNDING INFORMATION

The authors express their appreciation to the Princess Nourah Bint Abdulrahman University Researchers Supporting Project (PNURSP2026R213), Princess Nourah Bint Abdulrahman University, Riyadh, Saudi Arabia.

## CONFLICT OF INTEREST STATEMENT

None.

## ETHICS STATEMENT

None.

## Data Availability

Data not applicable, as no datasets were generated or analyzed during the current study.
